# Targeted Transcriptomics of Frog Virus 3 in Infected Frog Tissues Reveal Non-Coding Regulatory Elements and microRNAs in the Ranaviral Genome and Their Potential Interaction with Host Immune Response

**DOI:** 10.3389/fimmu.2021.705253

**Published:** 2021-06-17

**Authors:** Yun Tian, Collins N. Khwatenge, Jiuyi Li, Francisco De Jesus Andino, Jacques Robert, Yongming Sang

**Affiliations:** ^1^ Department of Agricultural and Environmental Sciences, College of Agriculture, Tennessee State University, Nashville, TN, United States; ^2^ Department of Microbiology and Immunology, University of Rochester Medical Center, Rochester, NY, United States

**Keywords:** frog virus 3, ranavirus, transcriptome, *cis*-regulatory elements, microRNA, interferon signaling

## Abstract

**Background:**

Frog Virus 3 (FV3) is a large dsDNA virus belonging to Ranaviruses of family *Iridoviridae*. Ranaviruses infect cold-blood vertebrates including amphibians, fish and reptiles, and contribute to catastrophic amphibian declines. FV3 has a genome at ~105 kb that contains nearly 100 coding genes and 50 intergenic regions as annotated in its reference genome. Previous studies have mainly focused on coding genes and rarely addressed potential non-coding regulatory role of intergenic regions.

**Results:**

Using a whole transcriptomic analysis of total RNA samples containing both the viral and cellular transcripts from FV3-infected frog tissues, we detected virus-specific reads mapping in non-coding intergenic regions, in addition to reads from coding genes. Further analyses identified multiple *cis*-regulatory elements (*CREs*) in intergenic regions neighboring highly transcribed coding genes. These *CREs* include not only a virus TATA-Box present in FV3 core promoters as in eukaryotic genes, but also viral mimics of *CREs* interacting with several transcription factors including CEBPs, CREBs, IRFs, NF-κB, and STATs, which are critical for regulation of cellular immunity and cytokine responses. Our study suggests that intergenic regions immediately upstream of highly expressed FV3 genes have evolved to bind IRFs, NF-κB, and STATs more efficiently. Moreover, we found an enrichment of putative microRNA (miRNA) sequences in more than five intergenic regions of the FV3 genome. Our sequence analysis indicates that a fraction of these viral miRNAs is targeting the 3’-UTR regions of *Xenopus* genes involved in interferon (IFN)-dependent responses, including particularly those encoding IFN receptor subunits and IFN-regulatory factors (IRFs).

**Conclusions:**

Using the FV3 model, this study provides a first genome-wide analysis of non-coding regulatory mechanisms adopted by ranaviruses to epigenetically regulate both viral and host gene expressions, which have co-evolved to interact especially with the host IFN response.

## Introduction

Frog virus 3 (FV3) is a large (~105 kb), double-stranded DNA (dsDNA) virus belonging to Ranaviruses of the family *Iridoviridae*, which consists of a group of emerging viruses infecting fish, amphibians, and reptiles (1, 2). FV3 infects amphibians at various life stages; whereas the infection is usually lethal in tadpoles, adult animals are more resistant and even become asymptomatic carrier following the infection. Hence, FV3 has been isolated from both sick and apparently healthy frogs in the wild and laboratory conditions (1–3). The association of FV3 with apparently healthy frogs indicates host-adaptive evolution for effective viral transmission and infection manifested at susceptible stages during the amphibian life cycle (1–3). This resembles the balance between deadliness and contagiousness exhibited by most successful viruses, which have effectively caused epidemics even pandemics in affected animals and humans (4). Increasing evidence suggests that Ranaviruses are important contributors of the catastrophic global amphibian declines, which pose emerging pressure on bio-ecological health and biodiversity (5–7). So far, FV3 acts as the most frequently reported iridovirus in infected anuran cases worldwide; it is widespread in wild amphibians and the only ranavirus detected in turtles in North America (5–8). Vilaça et al. (8) detected several FV3 lineages in wild amphibians in Canada, and these new FV3 isolates seem to have undergone genetic recombination with common midwife toad virus (CMTV) (8, 9). In this context, CMTV represents another ranavirus endangering amphibians and reptiles throughout Europe and Asia (8, 9). Owing to their prevalence and negative impact on many aquatic vertebrate species, more extensive studies of ranavirus biology at the genomic and molecular level are needed (1–9).

FV3 is the one of the best characterized models for ranaviral research, and previous studies using this virus have discovered features applicable to all iridoviruses, including the characterization of two-stage viral genome replication, phage-like hyper-methylated genomic DNA, temporal transcription of coding genes, and virus-mediated arrest of host immune response (10–14). Focused on coding genes, early studies had classically examined the expression of 47 viral RNAs and 35 viral proteins in FV3-infected fish cell lines, and designated them into immediate early, delayed early, and late genes expressed in a sequential fashion during the viral infection (10–12). Majji et al. (15) reported a first FV3 transcriptomic analysis of all putative annotated 98 coding genes (or open reading frames, ORFs) using microarray (15). They identified 33 immediate early (IE) genes, 22 delayed early (DE) genes, 36 late (L) viral genes, while seven genes remained undetermined (15). These previous transcriptomic studies were performed *in vitro* mostly using a model of fathead minnow (FHM) fish cells (10–12, 15). Thus, FV3’s transcriptomic information *in vivo* in infected amphibians under pressure from host various microenvironment and immune responses may provide important and more realistic information about ranaviral transcriptome. Furthermore, besides the 98 coding genes that occupy about 80% of FV3’s genome, there are about 50 intergenic regions from 20 to 900 nt long spanning the remaining ~20% of FV3’s genome. The potential regulatory property and transcription of these non-coding genomic regions is largely unknown. Given the relatively small size of viral genomes (even for large DNA viruses), it is reasonable to hypothesize that these intergenic regions in the FV3 genome exert a regulatory role underlying viral gene expression and virus-host interaction, especially at the epigenetic level (16, 17).

The best-characterized core promoter in eukaryotic genes contains a TATA-Box, which is located at the positions −25 and −30 from the transcription start site (TSS). The TATA-Box is recognized by the TATA-binding protein (TBP) in a complex of several other transcription factors (TF), which recruits the RNA polymerase II (pol II) to initiate transcription process (18). Viruses rely on cellular metabolism for completing their infection cycle. Viral genes, thus, adopt similar *cis*-regulatory elements (*CREs*) for interacting with host transcription machinery and orchestrating viral and host gene expression (16). For example, in human herpes simplex viruses (HSV), a recent study detected the binding sites for TBP, pol II, and a viral ICP4 protein on the promoter regions of representative immediate early (IE), early (E), and late (L) genes, and relevant *CRE*-TF interaction to mediate associated HSV gene expression in a function of time post-infection (19). Various promoter elements have also been examined in other large dsDNA viruses of *Poxviridae, Asfarviridae; Phycodnaviridae* and *Iridoviridae* (20). Studies of viral gene promoters in iridoviruses have mainly used FV3 and only focused on a few genes. A *cis*-element with 23 bp core region at 78-bp upstream of a major FV3 IE gene encoding ICP-18 (a.k.a, ICR-169, encoded by FV3gorf82R), was shown to interact with a FV3 protein (and potentially other cellular transcription factors) critical for transcription of ICP-18 gene (21). Additional analysis of the promoter region for another IE gene encoding ICP-46 (a.k.a ICR489, encoded by FV3gorg91R) detected no similar *CRE* (22). This lack of similarity between the two IE gene promoters indicated that the temporal regulation of IE genes is diverse. Furthermore, other *CRE* elements including those containing ‘TATA’, ‘CAAT’, and ‘GC’ motifs were identified in the ICP46 gene promoter, like to those of typical eukaryotic gene promoters (21–23). Other studies of three Bohle iridovirus genes —two early (*ICP-18* and *ICP-46*) and one late (major capsid protein [MCP]) identified conservative *CRE* motifs located 127 to 281 bp upstream of the transcription start site (TSS), and other ones located within 30 bp proximity to the TSS (21–24). While these studies provide a good first step, a more extensive analyses of promoter and relevant *cis-trans* interaction are imperative for understanding the temporal expression and transcriptomic profile of ranaviral genes, and for progressing in comparative studies of large dsDNA viruses (16, 20).

Viruses have evolved various strategies to evade host immune responses. In addition to the commonly studied antagonistic role exerted by viral proteins, multiple families of viruses, particularly DNA viruses, also encode regulatory microRNA (miRNA) species (25). miRNAs are small non-coding RNAs acting as RNA silencing and post-transcriptional regulators of gene expression by targeting primarily 3’-UTR regions of cellular transcripts. Virus-derived miRNAs (v-miR) potently act on either host or virus transcripts, and have been shown to be critical in shaping host-pathogen interaction (26). A variety of v-miRs has been identified in different DNA viruses, and their role in viral pathogenesis is emerging. v-miRs can subvert host defense responses and mediate other cellular processes such as cell death and proliferation. Whether v-miRs are present in ranavirus and play a role in regulation of virus-host interaction is largely unknown (25, 26).

Along with recent virome studies and the identification of novel ranavirus isolates (8, 9), we performed a whole transcriptomic analysis (RNA-Seq) using total RNA samples containing both the viral and cell transcripts from FV3-infected frog tissues (27). The virus-specific transcriptome mapped authentic reads, which spanned the full FV3’s genome at ~10× depth (both positive and negative strands) in several infected tissue including intestine, liver, spleen, lung and particularly kidney. Focusing on viral coding genes, we previously profiled their differential expression in a virus-, tissue-, and temporal class-dependent manners. Further functional analysis based on transcriptomic detection unraveled some viral genes encoding hypothetical proteins that contain domains mimicking conserved motifs found in host interferon (IFN) regulatory factors (IRFs) or IFN receptors (27). The IFN system is a critical antiviral mechanism that has diversified during vertebrate evolution. The IFN system in most tetrapod species include three types of IFNs (type I, II, and III), which are classified mainly based on type-specific molecular signatures and recognizing receptors (28–30). The binding of an IFN ligand with its cognate receptor, thus, elicits a signaling cascade involving IFN receptors and various transcription factors such as IRFs and STATs (28–30).

Here, we report that in addition to reads mapping in the coding region, we also detected RNA-Seq reads that distributed in non-coding intergenic regions of both positive and negative strands the FV3 genome. Further analyses identified various non-coding regulatory *CREs* in these intergenic regions corresponding to transcriptomic profiles of the coding genes. These *CREs* include those similar to TATA-Box marking the core promoters of typical eukaryotic genes (18), and viral mimics of *CREs* interacting with various transcription factors including CEBPs, CREBs, IRFs, NF-κB, and STATs, which are critical for regulation of cellular immunity and cytokine responses in antimicrobial immunity (29, 31). Moreover, we discovered for the first time, an enrichment of putative viral miRNA sequences in more than five intergenic regions of FV3 genome. A variety of these viral miRNAs have the potential to target the 3’-UTR of *Xenopus* genes involved in antiviral IFN response, including those encoding IFN receptor subunits and IRFs (26). Collectively, using FV3 model, this study provides a first comprehensive genome-wide analysis of non-coding regulatory mechanisms acquired by ranavirus pathogens to epigenetically regulate both viral and host gene expressions.

## Materials and Methods

### Virus Stock Preparation, Cell Culture, and Animals

Two Frog virus 3 (FV3) strains, a wild type (FV3-WT) and an ORF64R-deprived strain (FV3-Δ64R), were used. The virus preparation and animal infection were conducted as previously described (13, 27, 32). In brief, fathead minnow (FHM) cells (ATCC^®^ CCL-42) or baby hamster kidney (BHK) cells (ATCC^®^ CCL-10) or a kidney A6 cell line (ATCC^®^ CCL-102) were maintained and used for propagation and titration of FV3 virus stocks. Virus stocks were purified and the virus load was assessed by plaque assays in the BHK or A6 cells. Outbred specific-pathogen-free adult (1-2 years old) frogs were obtained from the *X. laevis* research resource for immunology at the University of Rochester (http://www.urmc.rochester.edu/mbi/resources/xenopus-laevis/).

### Ethics Statement, Animal Infection and Tissue Collection

Animal handling procedures were approved and performed under strict laboratory and University Committee on Animal Resources (UCAR) regulations (approval number 100577/2003-151). Adult frogs with the comparable Age/body-weight were randomly allotted into mock controls and infected groups (n = 5/group). Animal infections were conducted by intraperitoneal (i.p.) injection with FV3-WT (at 1 × 10^6^ PFU/each) or FV3-Δ64R (at 1 × 10^6^ PFU/each) virus in 100-μl amphibian phosphate-buffered saline solution (APBS) or only APBS for mock controls. At 0, 1, 3, and 6 days postinfection (dpi), animals were euthanized and indicated tissues were sampled and pairwise allotted for classical viral titration and gene expression analyses, and the samples of 3 dpi were cryopreserved for further transcriptomic analysis as described (13, 27, 32, 33).

### DNA/RNA Extraction and PCR/RT-PCR Assays

Total RNA and DNA were isolated from frog cells or tissues using a TRIzol reagent (Invitrogen) for PCR-based assays or a column-based RNA/DNA/protein purification kit (Norgen Biotek, Ontario, Canada) for transcriptomic analysis. RNA concentration and integrity were examined with a NanoDrop 8000 spectrometer (NanoDrop, Wilmington, DE) and an Agilent 2100 Bioanalyzer (Agilent Technologies, Santa Clara, CA) to ensure RNA samples with A260/A280>1.8 and RNA integrity number (RIN) >7.0 qualified for construction of sequencing libraries (27, 32, 33).

Quantitative PCR (qPCR) or qRT-PCR assays were conducted as described (29, 33). In brief, 150 ng/reaction of DNA templates were used to measure FV3 gene copies based on detection of FV3gorf60R, which encodes a viral DNA polymerase II (Pol II), in an ABI 7300 real-time PCR system and PerfeCta SYBR green FastMix, ROX (Quanta) (29, 33). For qRT-PCR analyses, assays were performed in a 96-well microplate format using a QuantStudio™ 3 Real-Time PCR System (Thermofisher) with the validated primers. Reactions were formed with a SYBR Green RT-PCR kit (Qiagen, Valencia, CA) with 500 ng of total RNA in a 20-μl reaction mixture. Specific optic detection was set at 78°C for 15 s after each amplification cycle of 95°C for 15 s, 56–59°C for 30 s and 72°C for 40 s. Cycle threshold (Ct) values and melt curves were monitored and collected with an enclosed software. Relative gene expression was first normalized against Ct values of the housekeeping gene (GAPDH) for relative expression levels, and compared with the expression levels of control samples for stimulated regulation if needed (29, 32, 33).

### Transcriptomic Analyses (RNA-Seq)

RNA sample and RNA-Seq sequencing library preparation were performed using the Illumina Pipeline (Novogene, Sacramento, CA) as previously described (27). For RNA-Seq, approximately 40 M clean reads per sample were generated for sufficient genome-wide coverage. The clean reads were assembled and mapped to the Reference genome/transcripts of *X. laevis* or FV3 virus through Xenbase (http://ftp.xenbase.org/) or NCBI genome ports (ftp://ftp.ncbi.nlm.nih.gov/genomes/all/GCF), respectively. Data of virus-targeted transcriptome was reported here. The workflow of RNA-Seq analysis, bioinformatics software used, and some exemplary data to show general quality and comparability of the transcriptome data was schematically shown and previously reported (27). Differentially expressed genes (DEGs) between two treatments were called using DeSeq and edgeR packages and visualized using bar charts (FPKM) or heatmaps (Log2 fold ratio) as previously described (27). The transcriptomic dataset was deposited in the NIH Short Read Archive (SRA) linked to a BioProject with an accession number of PRJNA705195.

### FV3-Genome Intergenic Regions and Associated *CRE* Analyses

The sequences of 51 intergenic regions between coding ORFs (including the 5’- and 3’-UTR regions of the viral genome) were extracted from FV3’s reference genome (GenBank accession number: NC_005946.1). The sequences were aligned using the multiple sequence alignment tools of ClustalW or Muscle through an EMBL-EBI port (https://www.ebi.ac.uk/). Other sequence management was conducted using programs at the Sequence Manipulation Suite (http://www.bioinformatics.org). Sequence alignments were visualized using Jalview (http://www.jalview.org) and MEGAx (https://www.megasoftware.net). Two programs/databases were used to confirm each other for the major *CRE* detection. The *CREs* (and corresponding binding TFs) in intergenic regions were examined against both human/animal TFD Database using a program Nsite (Version 5.2013, at http://www.softberry.com). The mean position weight matrix (PWM) of key *cis*-elements in intergenic regions were examined and calculated using PWM tools through https://ccg.epfl.ch/cgi-bin/pwmtools, and the binding motif matrices of examined TFs were extracted from MEME-derived JASPAR CORE 2020 vertebrates or JASPAR CORE 2018 vertebrates clustering affiliated with the PWM tools (34).

### Comparative *CRE*-Analysis of Intergenic Regions Immediately Upstream of Top-Ranked Highly Expressed FV3 Genes

FV3’s coding genes were categorized based on their temporal classes into immediate early (IE), delayed early (DE), and late (L) viral transcripts as previously designated. The expression levels of individual FV3 ORF coding genes were determined as averages across all samples to demonstrate the differential expression using the transcriptomic data. The relative expression order across and within each temporal gene classes was sorted. The intergenic regions immediately upstream of top-ten highly expressed FV3’s coding genes in each temporal class were extracted to perform PWM analyses as described above, and were compared to overall scores of all intergenic regions. The comparative analyses were broadly performed against various *CRE* types/clusters, but focused on those potently interacting with vertebrate transcription factors critically in antiviral immune regulation including CEBPs, CREBs, IRFs, NF-κB2-like, and STAT1-like transcription factors (31, 34).

### FV3-Genome Intergenic Regions and Associated Viral miRNA (v-miR) Analyses

The miRNA prediction and RNA structure prediction were analyzed using a findMiRNA and FoldRNA programs, respectively, through an online bioinformatic suite at http://www.softberry.com. The miRNA target prediction on the 3’-UTR of various *Xenopus* genes were performed using three RNA analysis programs through an online BiBiServ Service (https://bibiserv.cebitec.uni-bielefeld.de/). The sequences of 3’-UTR regions and information about alternative transcripts of *X. laevis* genes/transcripts were extracted from the gene annotations at Reference genome/transcripts of *X. laevis* or FV3 virus through Xenbase (http://ftp.xenbase.org/) and NCBI genome ports (ftp://ftp.ncbi.nlm.nih.gov/genomes/all/GCF). The locations and sequences of all predicted v-miR are listed in **Supplemental Excel Sheet**, and the GenBank accession numbers of analyzed genes/transcripts are listed in indicated tables.

### Transcriptomic Validation of miRNA Regulatory Effect on *Xenopus* Gene Targets in IFN Signaling

Due to the enrichment of predicted v-miR target sites on the 3’-UTR of *Xenopus* IRF and IFN receptor genes, transcriptomic analyses of *X. laevis* mRNA encoding various IRF and IFN receptor gene families to show the differential expression of these genes was compared between FV3-Δ64R- and FV3-WT-infected tissues. Wherein, some intergenic regions containing putatively responsible v-miR were demonstrated to transcribe differentially between these two FV3 strains. Particularly, several representative v-miR were synthesized and transformed into *X. laevis* A6 kidney cells to evaluate RNA interference effect against *Xenopus* IRF and IFN receptor genes. The small interfering RNA (siRNA) identical to representative v-miR sequences were synthesized and transformed as previously described (35). In brief, the sense and antisense sequences of the siRNA were synthesized at IDT (Coralville, Iowa) together with an AlexaFluor-488 (AF488) labeled scramble siRNA, which was designed to serve as control siRNA and allow transfection optimization. A6 cells were cultured as described in a 24-well plate and transfected with Oligofectamine (Invitrogen to attain >90% transfected ratio as estimated by the AF488-scramble siRNA (35). Forty-eight hours after siRNA transfection, cells in different wells were collected for RNA extraction and gene specific RT-PCR was used to quantify the expression of target genes as described above (27, 33). RNA samples used for RT-PCR assays were treated with RNase-free DNase I (NEB) to remove potential DNA contamination (29, 33).

### Statistical Analysis

Statistical analysis was completed using the SAS package (Company information)?. One-way analysis of variance (ANOVA) and Tukey’s *post hoc* test, as well as a two-sample *F* test was applied for significant evaluation between samples/treatments. A probability level of *p*<0.05 was considered significant (27, 32, 33).

## Results and Discussions

### Percent of Reads Mapped to Functionally Different Regions on FV3 Genome

**Figure 1:**
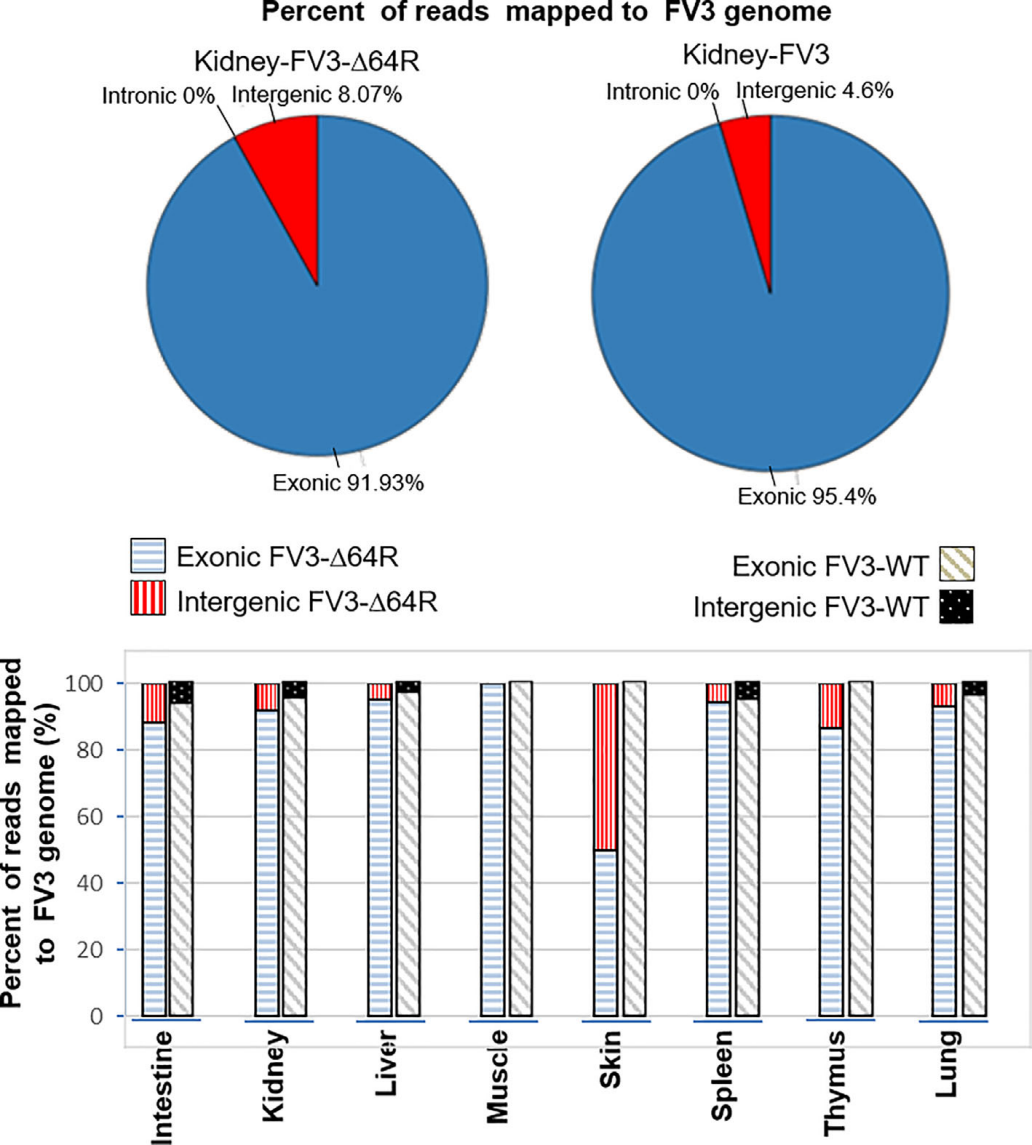
Percent of reads mapped to functionally different regions on FV3 genome. The FV3 genome regions are functionally classified as exons, introns, or intergenic regions based on annotation of the reference genome (NC_005946.1). As intronic regions (introns) are lacking in ranaviral coding genes, about 50 intergenic regions are interspersed between ORFs. The intergenic regions take about 20% of the FV3 genome with a length of 20-900 bp. Transcriptomic reads in most infected tissues are also remarkably mapped within these intergenic regions, indicating that these intergenic regions are transcribed and probably function as regulatory RNA species.

The FV3 genome regions are functionally classified into exons, or intergenic regions based on annotation of the reference genome (NC_005946.1). All FV3’s coding ORFs span about 80% of the genome sequence, and lack introns, i.e., intronless (27). In contrast, we extracted 51 intergenic regions that are intermediate between sequential ORFs, including the terminal 5’- and 3’-untranslational regions (UTRs) that are known to play important regulatory role in viral replication and gene expression. These ranaviral intergenic regions take about 20% of the FV3 genome with a length varying from 20 to 900 bp and an average length of 340 bp long. As expected, the majority of RNA-Seq reads (>90%), representing a significant coverage of the whole viral genome, mapped to coding regions in most infected tissues including the intestine, kidney, liver, spleen, thymus and lung (**Figure 1**). However, a careful examination of virus-specific reads in most infected tissues also detected ~5-10% authentic reads being specifically mapped on intergenic regions. This indicates that these intergenic regions in the FV3 genome are transcribed and probably function as regulatory RNA species. In addition, consistent with data previously reported for coding genes, the FV3-Δ64R mutant virus had also a general higher transcription of reads mapped to intergenic regions in most infected tissues (**Figure 1**) (27). This implies that the disruption of the FV3orf64R gene, which encodes a putative interleukin-1 beta convertase containing caspase recruitment domain (vCARD), may change the overall viral transcription dynamics, or result in accumulation of viral transcripts due to inefficient virus assembly process (36).

### Distribution of TATA-Box-like *cis*-Element in Intergenic Regions of FV3 Genome and Association With FV3’s Coding Gene Expression

**Figure 2:**
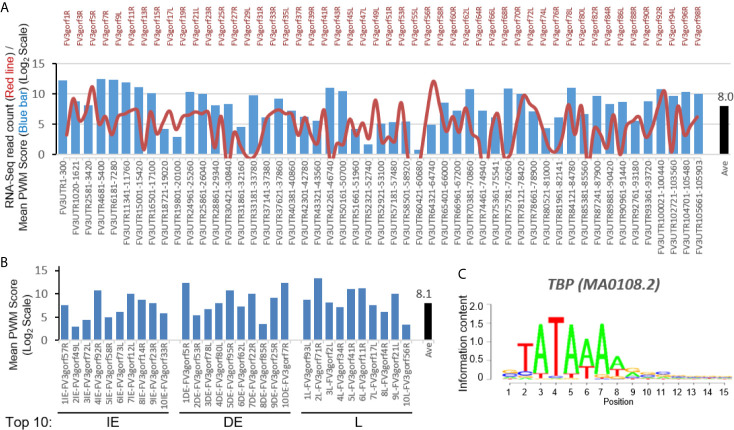
Transcriptomic comparison and distribution of TATA-Box-like *cis*-element in intergenic regions of the FV3 genome. **(A)** Line chart depicts cross-tissue averages of RNA-Seq reads differentially mapped to intergenic regions and almost all annotated FV3 coding ORFs labeled on the top. Note the X-Axis tick labels on the top for even-numbered ORFs (such as FV3gorf2L between FV3gorf1R and FV3gorf3R) are omitted due to the space limitation. Bar chart depicts the position weight matrix (PWM) scores of the TATA-box, a *cis*-regulatory element (CRE) marking core promoters of eukaryotic genes significantly detected across all FV3-genome intergenic regions (labeled as FV3UTR start-end nt position along the FV3 reference genome). **(B)** Mean PWM scores of TATA-box CRE in FV3 intergenic regions that are intermediately upstream of top-ten highly expressed FV3 coding genes (ORFs) in each temporal class of immediate early (IE), delay early (DE), or late (L) genes as revealed by transcriptomic analyses. Mean PWM scores were calculated using tools at https://ccg.epfl.ch/pwmtools/pwmscore.php. In both **(A)** and **(B)**, the cross-panel mPWM scores of the TATA-box CRE is averagely (Ave) shown as data-labeled black bar at the right. **(C)** The matrix of TATA-box that interacts with a transcription factor of TATA-box binding protein (TBP) is from MEME-derived JASPAR CORE 2020 vertebrates affiliated with the PWM tools.

To reveal *cis*-regulatory role of intergenic regions on expression of coding genes, we first searched for putative viral TATA-box equivalent. In eukaryotic genes, the TATA-box is a *cis*-regulatory element (*CRE*) marking the core promoters. To identify a putative viral TATA-like box we used a software based on an evaluating score system of position weight matrix (PWM) used for vertebrate *CREs* (18, 19). The bar chart in **Figure 2A** shows that a significant score (pseudo-weight value <0.0001 as defaulted in the system) for putative FV3 TATA-box-like was detected in all intergenic UTR sequences including two terminal 5’- and 3’-UTR regions. The location of these putative TATA-Box-like *CREs* are at 11-470 nt (overall average at 190 nt) ahead of the TSS of downstream associated coding genes (**Supplemental Excel Sheet**). These results from a bulk study are consistent with previous single promoter characterization of a few genes in FV3 and Bohle iridovirus, where *CRE* motifs were found located 127 to 281 bp upstream of the TSS (24). The average PWM score of TATA-Box *CRE* across all intergenic regions was 8.0 (Log_2_Unit) with most scores higher than 5.0, which is close to the median value across PWM scores of multiple *CREs* executed in this study. The line chart in **Figure 2A** illustrates the transcriptomic average of all 98 coding genes annotated on the FV3 reference genome (27). Careful comparison did not show obvious positive correlation between higher PWM scores of TATA-Box-like in intergenic regions and increased expression of associated coding genes. A similar PWM score at 8.1 was obtained by executing the PWM evaluation for FV3 genes exhibiting top-ten ranked transcribing levels in different temporal classes (**Figure 2B**) (27). This suggests that although the putative TATA-Box *CRE* in intergenic regions may function to recruit vPol II through binding of the transcription factor TBP and signifies the core-promoter regions, it is not the only determinant (**Figure 2C**) (18). Rather these putative intergenic TATA-Box *CRE* are likely to cooperates with other intergenic *CREs* to induce relative expression levels of associated genes in the virus-host interaction (18, 19).

### Evolutionary Relevance of Predicted FV3 Intergenic CREs Binding to Immuno-Regulatory Transcription Factors

**Figure 3:**
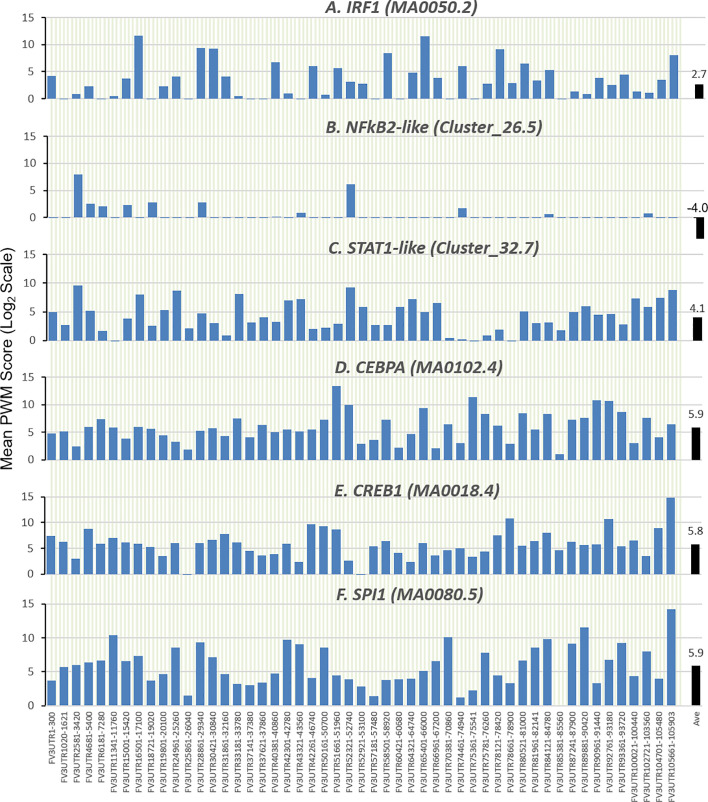
Comparison of position weight matrix (PWM) scores of key *cis*-regulatory elements (CREs) detected in FV3-genome intergenic regions, and that interact with vertebrate transcription factors potently in immune regulation. Shown are mean PWM scores of CREs in FV3 intergenic regions that were significantly detected to bind **(A)** IRF-like, **(B)** NF-κB2-like, **(C)** STAT1-like, **(D)** CEBP-like, **(E)** CREB-like, and **(F)** PU.1 (a.k.a. SPI1) transcription factors. Mean PWM scores were calculated using tools at https://ccg.epfl.ch/pwmtools/pwmscore.php with CRE Matrices (indicated by Matrix or Cluster numbers, and schematics in **Figure 4**) are from MEME-derived JASPAR CORE 2020 vertebrates or JASPAR CORE 2018 vertebrates clustering affiliated with the PWM tools. The genome-wide mPWM scores across all intergenic regions for each CRE are averagely shown (Ave) as data-labeled black bars at the right for overall comparison. CEBP, CCAAT enhancer binding protein beta; CREB, cAMP-response element binding protein; IRF, interferon regulatory factor; NF-κB, Nuclear factor-κB; SPI1 or PU.1, a TF binding PU-box, a purine-rich DNA sequence; and STAT, signal transducer and activator of transcription.

**Figure 4:**
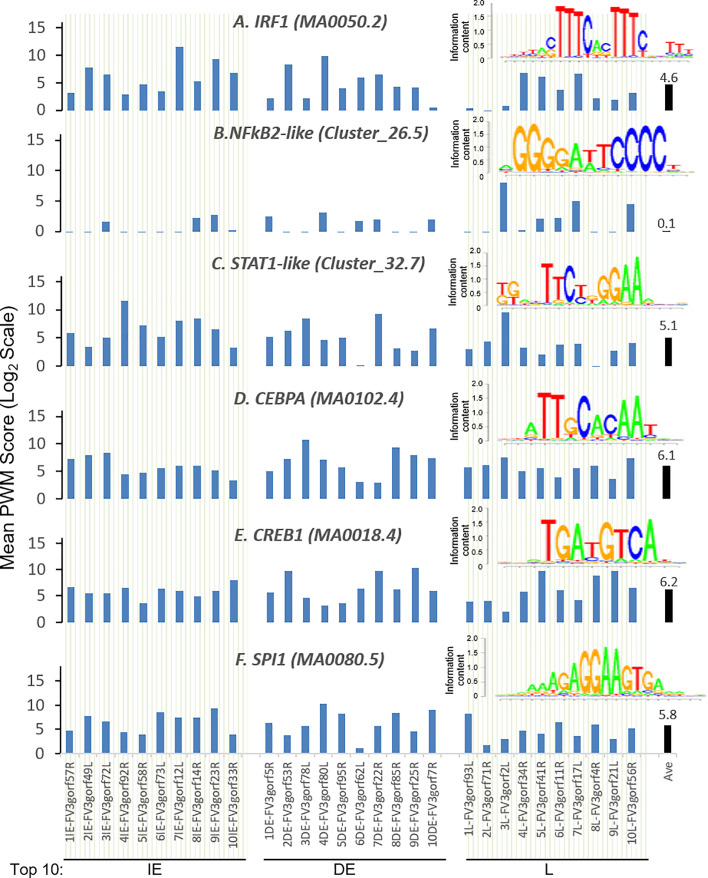
Intergenic regions immediately upstream of highly expressed FV3 genes serve as putative core promoters with enhanced capacity to bind vertebrate transcription factors of **(A)** IRFs, **(B)** NF-κB2-like, and **(C)** STAT1-like, but not much enhanced for **(D)** CEBPA, **(E)** CREB1, and **(F)** SPI1 transcription factors. Shown are mean PWM scores of *cis*-regulatory elements (CREs) in FV3 intergenic regions that are immediately upstream of top-ten highly expressed FV3 coding genes (ORFs) in each temporal class of immediate early (IE), delay early (DE), or late (L) genes. Mean PWM scores were calculated using tools at https://ccg.epfl.ch/pwmtools/pwmscore.php with CRE Matrices are from MEME-derived JASPAR CORE 2020 vertebrates or JASPAR CORE 2018 vertebrates clustering affiliated with the PWM tools. The cross-panel average mPWM scores (Ave) of each CRE are shown as data-labeled black bars at the right for overall comparison. Abbreviations of TFs are as in **Figure 3**.

**Figure 5:**
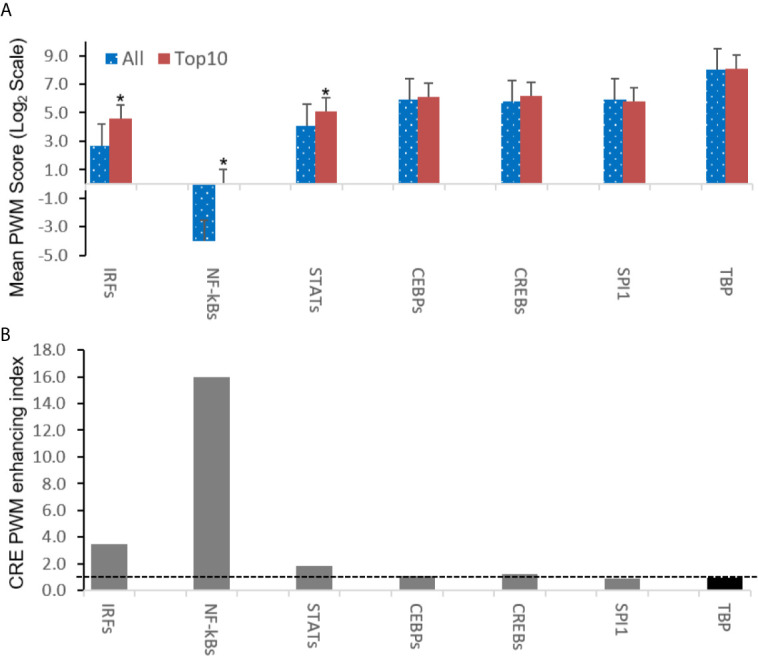
Intergenic regions immediately ahead of highly expressed FV3 genes containing *cis*-regulatory elements (CREs) exhibit higher likelihood of binding vertebrate IRFs, NF-κB2-like, and STAT1-like transcription factors. **(A)** Shown are overall averages of PWM scores per compared CREs in all FV3 intergenic regions (All) and those are immediately upstream of top-ten highly expressed FV3 coding genes (Top10) in each temporal class of immediate early (IE), delay early (DE), or late (L) genes as revealed by transcriptomic analyses. Mean PWM scores were calculated as in previous figures. *p < 0.001 and n = 10, compared to the All group. **(B)** The CRE PWM enhancing index was adopted to compare fold changes of mean PWM scores between the Top10 and All groups after normalization with the PWM evolution of TATA-box between the two groups as baseline (indicated by the dash line). Abbreviations of TFs are as in **Figure 3**.

Further analysis detected the presence of multiple types of viral *CRE* mimics (*v-CREs*) in FV3 genome intergenic regions. We focused our interest on *CRE* families that are critical in regulation of amphibian antiviral immunity. These *v-CREs* include those predicted to interact with transcription factors (TFs), such as the IRF and STAT families that critically mediate cytokine- and IFN-dependent signaling. Among these factors, NF-κB-like and PU.1 (a.k.a. SPI1) regulate inflammation, whereas other like the CEBP and CREB families control immune cell proliferation and activation (37–41). **Figure 3** shows the distribution *v-CREs* that have likely evolved to interact with representative TFs critical for regulating antimicrobial immunity as aforementioned. Besides *v-CRE* showing a significant binding score for IRF1, most intergenic regions also exhibit conserved *v-CREs* with comparable PWM scores that can bind IRF2 IRF5 and IRF6 (**Figure 3** and **Supplemental Excel Sheet**). In contrast, only a portion (a third to a half) of intergenic regions contain *v-CREs* with a high PWM binding score (>2 Log_2_Unit) for other IRFs. Similarly, *v-CREs* with significant prediction for binding members of the STAT family were detected in almost all intergenic regions and for all vertebrate STAT members with average PWM scores between 2.0-6.0 log_2_Unit in an increasing order of STAT1(2.0)<STAT4≈STAT6(4.0)<STAT3(5.0)<STAT2≈STAT5a/b(6.0) (**Supplemental Excel Sheet**). Most intergenic regions also contained *v-CREs* with predicted binding to members of the CEBP, CREB and SPI1 families with average PWM scores close to 6.0 log_2_Unit (**Figure 3** and **Supplemental Excel Sheet**). We further extracted the sequences of these *v-CREs* from the intergenic regions immediately upstream of the top-ten ranked highly expressed FV3 genes of IE, DE and L temporal classes (**Figure 4**). Similar to the TATA-box-like in the promoter region of TBP, *v-CREs* located in intergenic regions associated with these top-ranked highly expressed genes exhibit significant PWM scores for CEBP, CREB and SPI1. Remarkably, *v-CREs* for IRFs, STATs and especially NF-κB seem to have been enhanced their PWM index to interact with relevant TFs in the intergenic regions ahead of the top-ranked viral genes (**Figures 4** and **5**). Notably, although the *v-CRE* for NF-κBs has a very low PWM score across most intergenic regions (**Figures 3** and **4**), we detected a dramatic enhancement of the *v-CRE* for NF-κB2 ahead of some top-ranked highly expressed viral genes (**Figure 5**). The NF-κB transcription factors comprise NF-κB1 and NF-κB2, which are activated by canonical or non-canonical signaling pathways, respectively (41). In addition to the canonical pathway activated by various pathogens and inflammatory cytokines, recent studies have discovered that dysregulation of non-canonical NF-κB2-mediated signaling is associated with severe immune deficiencies and various autoimmune diseases (41). The enhancement of *v-CRE* predicted binding to NF-κB2 in priming highly expressed viral genes, thus, may confer a potential antagonism attenuating host inflammatory and autoimmune responses at the epigenetic level (27, 41). In this context, the enhancement of *v-CREs* binding to IRF and STAT transcription families may perturb host cytokine responses and particularly IFN-mediated antiviral signaling, which have been observed in our previous studies in terms of suppression of IFN signaling in FV3-infected amphibians (13, 32, 33). Recent studies have also highlighted the immunopathological effect of persistent IFN production during chronic viral infections, as well as autoimmune and inflammatory diseases. In these cases, IFN gene activation was sustained by chromatin remodeling through epigenetically recruiting IRF1, NF-κB and SPI1 transcription factors to the gene promoter region (27, 42). Data presented here about the *v-CRE* preservation and enhancement for SPI1 and IRFs/NF-κB, especially for highly expressed viral genes, may indicate molecular evolution of ranaviral intergenic regions in host/pathogen arm race with epigenetic regulation of the host IFN system (39–42).

### FV3 Intergenic Regions Are Enriched for Putative Regulatory microRNA Sequences

Micro RNAs (miRNAs) define a class of small (21-25 nt), non-coding regulatory RNA species discovered widely across biological kingdoms from bacteria to humans (43, 44). Micro RNAs are produced from typical hairpin-shaped precursors, and are involved in suppression of gene expression through specific ribonucleotide complementarity in the 3’-UTR of mRNAs to induce mRNA cleavage or translation repression (43, 44). In addition, the positive effect of miRNAs to activate target gene translation or transcription has been reported recently (44). Micro RNAs represent a major non-coding regulatory mechanism that shapes cellular transcriptome and is involved in microbe-host interaction (45). Given their small size and multi-targeting property, miRNAs are ideal epigenetic mechanism for viruses that have limited genome capacity (25, 26, 45). Indeed, diverse virus families, particularly DNA viruses, are capable of using host miRNA or even encode viral microRNAs. Virus-derived miRNAs (v-miR), which act on either host or virus transcripts, have been shown to be critical in shaping host-pathogen interaction. There is increasing evidence of their role in subverting host defense responses and mediating other cellular processes underlying antiviral immunity (25, 26, 45). However, we know little about ranavirus-derived v-miR and their potential mRNA targets in regulation of virus-host interaction. In the following sections, we present data showing that intergenic regions of FV3 genome contain a wealth of miRNA-like sequences as determined by the sequence and structure analyses of the precursor and relevant mature miRNAs (**Figure 2** and **Supplemental Excel Sheet**). These v-miR-containing clusters in FV3 genome are particularly enriched in five intergenic regions, which are marked as C, I, R, AF and AT to indicate their higher miRNA density and distribution ahead of several highly transcribed genes along the genome (**Figure 2**). Therefore, for the first time we reveal that a ranavirus genome, like other large DNA viruses, encode a series of miRNA especially using some intergenic non-coding sequences (43–45).

### Transcripts of the IFN Receptor Beta Subunits Emerge as Potential Major Targets of FV3-Derived miRNAs

**Figure 6:**
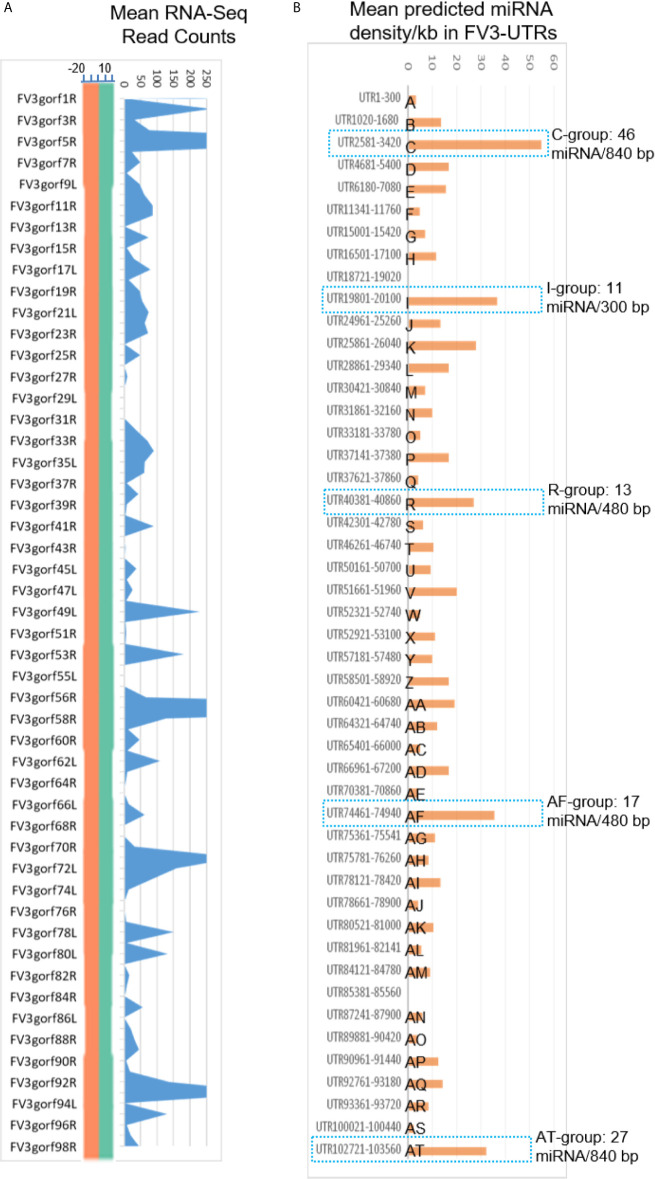
Comparison of transcriptomic and enrichment of putative microRNA (miRNA) sequences in intergenic regions of FV3 genome. **(A)** As line chart in **Figure 2**, mean RNA-Seq reads are differentially distributed among intergenic regions and almost all annotated FV3 coding ORFs. A distribution plot between the vertical Axis and gene labels, shows the median of read density (Log2 Unit) of mapped reads along the FV3 genome as in the FV3-Δ64R-infected kidney to show the full-genome coverage at both positive (green) and negative (orange) strand orientations. Transcription of the intergenic regions along the higher read density spanning the ORF coding genes is shown using the shaded blue curve indicating mean read counts across the eight infected tissues tested. **(B)** The prediction of miRNA-like sequences in most intergenic regions (marked as UTR start-end site along FV3 reference genome including the 5’- and 3’-untranslated regions), which are especially enriched in five regions (named as C, I, R, AF and AT per putative miRNA density/Kb) as marked using blue dash line. The sequence information of all predicted miRNAs is listed in **Supplemental Excel Sheet**. The miRNA prediction and target validation were performed using three RNA analysis programs through an online BiBiServ Service.

The vertebrate IFN system is constituted of three types of IFNs, i.e., type I, II and III IFNs, which exert diverse immune function initiated through the engagement of type-specific cognate receptors that comprise two subunits as of IFNAR1/2, IFNGR1/2, and IFNLR1/IL10RB, respectively (46). Amphibians have been recently characterized for their unique position in IFN molecular evolution and the complexity of their IFN system (29), as well as for the diversity of their IFN receptor genes (46). For examples, compared with one gene locus encoding each IFN receptor subunit in humans and mice, *Xenopus* genomes may contain two or more gene loci especially for the beta subunits of IFN receptors, and the increased complexity of relevant gene composition is observed particularly in *X. laevis* species that has an allotetraploid genome (47). In addition, mRNA transcripts for the beta subunits (ifnxr2 or il10rb, x = a, g, or l) of three type IFN receptors bear a much longer 3’-UTR as compared with their alpha subunit counterparts (ifnxr1, **Table 1** and unpublished data). Target analysis has revealed a significantly higher density of v-miR-targeted sites within the 3’-UTR of the beta subunit mRNAs than alpha subunit of all three types of IFN receptor genes, especially those for type I and type III IFN (**Table 1**). Further group assignation showed that most miRNAs predicted to target IFN receptor genes belong to four of the major five groups, i.e., C, R, AF and AT group (**Figure 6**). This implies that v-miRs derived from these four intergenic regions may have evolved to interfere with amphibian IFN signaling through targeting mainly genes encoding IFN receptor beta subunits. Despite little previous studies on ranaviral miRs, *Xenopus* miRNAs have been characterized and shown to be highly clustered within transcribing introns in the genome (48, 49). Using the miRNAs listed in the Xenbase catalog, target analysis against the 3’-UTR of IFN receptor genes also resulted in similar enrichment of miRNA target sites relevant to genes of the IFN beta subunits (Data not shown). These data collectively indicate that miRNAs serve as an important regulatory mechanism that can modulate IFN signaling by silencing the expression of ifnxr2 subunits (43–45, 48, 49). In turn FV3-derived miRs may use this transcriptional regulation to facilitate its pathogenesis (42). Nevertheless, whether certain miRNAs exhibit predicted activity on transcription of IFN responsive genes remains to be shown *in vivo*.

**Table 1:** Enrichment of predicted FV3 miRNA targeting sites in the mRNA 3-UTR regions of interferon receptors, especially the beta subunits.

mRNA (GenBank Acc. #)	3’-UTR length (kb)	Target sites/kb by predicted FV3 miRNA*	No. of FV3 miRNA /Group
Ifnar1.L (XM_018245928)	0.163	0	0
Ifnar1.S (XM_018248888)	0.406	2.46	1/1 (1AT)
Ifnar2.L (XM_018245430)	0.439	**84.28**	26/9 (11C, 4AF, 4AT,,…)
Ifnar2.S (NM_001095360)	2.305	**76.79**	69/14 (30C,15AT, 6R, 5AF,…)
Ifnar2.2S (XM_018248427)	0.495	**68.69**	27/6 (14C, 4R, 3AF, 3AT,…)
ifngr1.S (XM_018265300)	0.138	7.25	1/1 (1C)
ifngr2.L(XM_018245930)	0.656	25.91	16/5 (9C, 4AT, …)
ifngr2.S(XM_018248887)	1.241	**45.93**	42/7 (19C, 8AT, 4R, 4AF…)
ifnlr1.L (XM_018242320)	0.156	0.00	0
il10rb.L (XM_018245931)	0.438	25.11	11/6 (3C, 3AT, 2AF,…)
il10rb.S (NM_001093545)	0.955	**77.49**	42/12 (17C, 10AT, 4AF, …)
	Ave: 0.672	Ave: 37.63	

Acc., accession; Ave., average; kb, kilobase; UTR, untranslated region. *Numbers higher than the average are bold.

**Figure 7:**
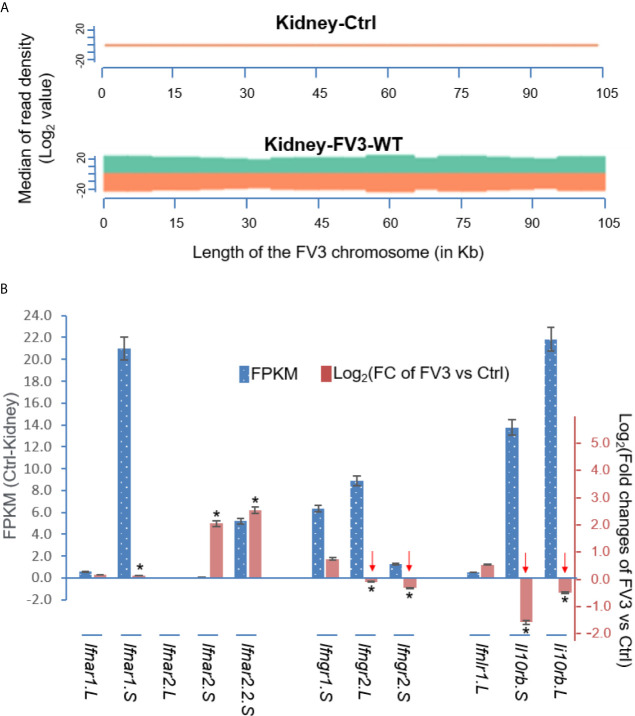
Transcriptomic analysis of the viral genome and *X. laevis* mRNA encoding interferon receptor subunits in the control (Ctrl) and FV3-infected kidney. **(A)** The virus-targeted transcriptome analysis shown as a distribution plot of mapped reads in FV3 genome (GenBank Accession No. NC_005946.1). The X-axis shows the length of the genome (in Mb, 0.105 Mb of FV3), and the Y-axis indicates the log_2_ of the median of read density. Green and red indicate the positive and negative strands, respectively. Note, no FV3 transcript read was obtained from the control (Ctrl) mock-infected kidney, and the full coverages of both positive and negative reads on the FV3 genome in the infected kidney. **(B)** Family-wide transcriptomic analysis of *X. laevis* mRNA encoding interferon receptor subunits for type I (ifnar1/2), II (ifngr1/2), and III (ifnlr1/il10rb) IFNs to show the differential expression of these IFN receptor genes in the kidney (Blue bars against the left Axis for FPKM, Fragments Per Kilobase of transcript per Million mapped reads) and regulated expression in FV3-infected kidney (Orange bars against the right Axis for Log2 fold changes). Note the significant reduction of the beta-subunits of type II and type III IFN receptors (indicated by red arrows), which may putatively result from a higher enrichment of the intergenic miRNA species as shown in **Table 1**. *p (FDR) < 0.05 relative to the control, n = 5.

**Figure 7A** presents a virus-targeted transcriptome analysis in the kidney from FV3-infected frogs. The kidney served as a primary site for FV3 replication and viral gene expression (27, 32, 33). Comparable amounts of RNA-Seq reads were detected from kidneys infected by either FV3-WT or FV3-Δ64R strains with mapped reads distributed along the full FV3 genome at a ~10× coverage depth. It is to note that no FV3 transcript read was obtained from the mock-infected control (Ctrl) samples, and that the full coverages of both positive and negative reads on the FV3 genome included intergenic regions (**Figures 6** and **7A**). As a point of comparison, **Figure 7B** shows transcriptomic data from the same infected tissues but focused on *X. laevis* mRNA transcripts that encode IFN receptor subunits for type I (ifnar1/2), II (ifngr1/2), and III (ifnlr1/il10rb) IFNs. The basal expression of these IFN receptor genes, as estimated by FPKM values (Fragments Per Kilobase of transcript per Million mapped reads) in the control kidney, shows a differential expression order: *ifnar1.S≈il10rb.L>il10rb.S>ifngr2.L≈ifngr1.S≈ifnar2.2.S>>others.* This observation raises several points about the intricated expression of IFN receptor genes in *X. laevis*: (1) In *X. laevis’s* allotetraploid chromosomes, both short (S) and long (L) subgenomes harbor actively expressed isoforms of IFN receptor genes (47); (2) Despite the existence of several genes encoding isoform for each IFN receptor subunit, only one gene was highly expressed. The only exception is for the two genes encoding the receptor beta subunit for type III IFNs (*il10rb.S and il10rb.L*), perhaps because il10rb is shared by IL-10 cytokine family (46, 50); and (3) genes encoding the alpha and beta Subunits of IFN receptors were expressed at a very different level.

We then compared differential expression of these IFN receptor genes between uninfected control and FV3-infected samples. Data indicate a significant reduction of gene expression of the beta subunits’ transcripts for the receptors of type II and III IFNs, but not type I IFNs (**Figure 7B**). Our interpretation of these data is that FV3 interferes with type II and III IFN signaling mainly through v-miRs encoded within the major five intergenic regions,. These v-miRs are likely to target the 3’-UTRs of host IFN receptor genes. However, the suppression of *ifnar1.S* and upregulation of *ifnar2.S* seemed not correlated to the v-miR-target prediction as shown in **Table 1**. This may indicate an inefficient RNA repression (or unusual activation effect) of the predicted anti-*ifnar2.S* v-miRs and a v-miR-independent suppression of *ifnar1.S* that warrants further investigation (**Figure 7B**) (43, 44).

**Figure 8:**
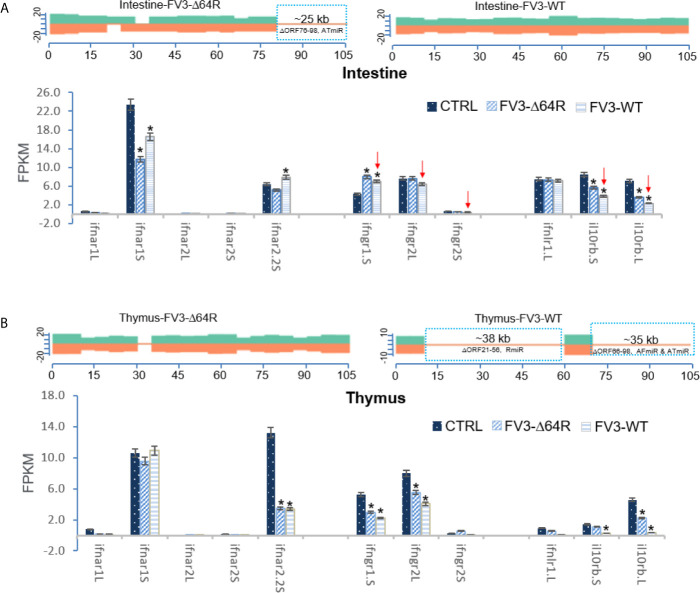
Transcriptomic comparison of the viral genome and *X. laevis* mRNA encoding interferon receptor subunits in the mock, FV3-Δ64R, and FV3-WT infected intestine **(A)** and thymus **(B)**. The distribution plots of mapped reads alone FV3 genome (GenBank Accession No. NC_005946.1) were shown as in **Figure 3**. Partial coverages of the viral genome were determined forFV3-Δ64R-infected intestine, and for both FV3-WT and FV3-Δ64R in the infected thymus. Comparative alignments showed that transcripts of some ORFs and miRNA-enriched intergenic regions were defective (labeled and framed using blue line) as compared between two virus strains. Red arrows indicate further repression of some IFN-receptor genes corresponding to potential higher expression of respective miRNA by FV3-WT in the intestine. The putative miRNA-mediated repression of IFN receptor genes is not detected in FV3-Δ64R infected thymus. Abbreviations and gene accession numbers are listed in **Table 1**. *p (FDR) < 0.05 relative to the control, n = 5.

Our analysis of RNA-Seq viral reads indicates a partial coverage of the FV3-Δ64R-FV3 genome in infected intestine and the thymus compared to wild type FV3. Aligned estimation shows that transcripts of some ORFs and miRNA-enriched intergenic regions are defective (**Figure 8**). Further repression of some IFN-receptor genes corresponding to potential higher expression of respective miRNA by FV3-WT was observed in the intestine. However, there was a lack of putative *v-miR-*mediated reduction of IFN receptor genes in FV3-Δ64R infected thymus compared to FV3-WT, where no transcribing activity of R-, AF- and AR-group miRs was detected. This suggests a tissue- and virus strain-dependent expression of v-miRs and RNA interference on host gene targets (27). Notably, the disruption of the FV3gorf64R gene encoding vCARD protein in FV3-Δ64R recombinant virus may alter viral transcription activity of intergenic regions including the v-miR clusters (36).

### FV3-Derived miRNAs May Have Evolved to Target Transcripts of *Xenopus* IFN Regulatory Factors

Interferon regulatory factors (IRFs) are a family of transcription factors that comprise about 10 homologous members (IRF1-9) in tetrapods (51). As studied in humans and mice, IRFs are key modulators of immune processes involving Toll-like receptor (TLR)- and IFN-dependent host responses (39, 51). Tetrapod IRFs are phylogenically assigned into five functional subgroups: IRF1&2, IRF3&7, IRF4&8, IRF5&6, and IRF9 (39, 51). Functionally, IRF1, considered as an ancestral IRF, has emerged to broadly mediate IFN-dependent inflammation and epigenetic regulation in monocytes and macrophages (39, 52). IRF1 and IRF2 also promote Th1 immune responses (39). IRF3 and IRF7 are activated by various signaling pathways leading to IFN production in the scenario of antiviral immunity (39, 46). IRF4 and IRF8 are highly expressed in lymphoid and myeloid lineages, where they regulate B cell development and Th cell differentiation (39, 53). For IRF5 and IRF6, the former is critical in control of inflammation mediated by macrophages and neutrophils; while IRF6 regulates epithelial barrier function and TLR-mediated inflammation therein (39, 51, 54, 55). IRF9 together with STAT1 and STAT 2 form a tripartite ISGF3 complex, which is criti-cal for signal transmission to both type I and III IFNs (39, 46). We also identified a fish IRF10 ortholog in *Xenopus*. The fish IRF10 shares gene synteny with IRF1 but functionally serves as a negative regulator for IFN production to avoid excessive immune response (56). Collectively, due to the crucial role of IRFs in antiviral signaling, the balance between the fine-tuning of IRF expression and viral antagonism capable of disarming IRF-mediated signaling, determines the pathogenesis and outcome of infection (39, 57). **Table 2** list the current IRF gene/transcript annotation on *X. laevis* genome. Compared with the genes/transcripts for IFN receptors, many *Xenopus* IRF transcripts have 3’-UTRs longer than 1.0 kb (averagely 0.862 vs 0.672 kb for IFN receptor transcripts in **Table 1**). However, a low density of putative v-miR targeting sites was detected within most 3’-UTRs of *Xenopus* IRF transcripts, except *irf5.S* and *irf10.L* that have a higher density around 50 per kb. Additionally, v-miRs predicted to target 3’-UTRs of IRFs were distributed widely in more intergenic regions than the five major intergenic regions containing putative v-miRs targeting transcripts of IFN receptors (**Table 2**). It is, therefore, possible that v-miRs derived from FV3’s intergenic regions target less intensively IRFs than IFN receptor transcripts. However, some IRF members including *Xenopus* irf1/2, irf5 and especially irf10 may still be selectively targeted. These genes have been mainly associated with immune regulation that is less studied in other animal species and remain uninvestigated in amphibians (39, 51–56). Interestingly, we have not detected any enrichment of v-miR-targeting sites in the 3’-UTR of transcripts encoding socs1.L and socs1.S, two TFs mediating negative regulation of IFN signaling in humans and mice (39, 46). The evidence indicating a target-site enrichment on some IRF transcripts by v-miRs suggests that FV3 and its v-miRs provide a good system for a cross-species examination of the immunomodulatory role of these understudied IRF homologs including irf1, irf2, and especially irf10 in *Xenopus* (39, 51).

**Table 2:** Distribution of predicted FV3 miRNA targeting sites in the mRNA 3-UTR regions of interferon regulatory factors (irfs).

mRNA (GenBank Acc. #)	3-UTR length (kb)	Target site/kb by predicted FV3 miRNA*	No. of FV3 miRNA /Group
irf1.L (NM_001089781)	1.038	**32.8**	34/8 (19C, 4R, 3D, 3AB, …)
irf1.S (NM_001092119)	1.152	**27.8**	32/8 (18C, 5R, 3AF, 2D…)
irf2.L (XM_018248817)	1.019	**29.4**	30/6 (17C, 6R, 3AB, 2AF...)
irf3.L (NM_001086119)	0.709	12.7	9/6 (2C, 2D, 2AF…)
irf3.S (XM_018228156)	0.480	18.8	9/5 (4C, 2D, 1E, 1AB, 1AF)
Irf4.S (XM_018269454)	0.496	0.0	0
irf5.L (NM_001094596)	0.353	**25.5**	9/3 (7C, 1AB, 1AF)
irf5.S (XM_018255680)	0.367	**49.0**	18/5 (9C, 4AB, 2D…)
irf6.2L(NM_001087746)	0.506	4.0	2/2 (1D, 1V)
irf6.S (NM_001091876)	0.910	6.6	6/4 (3C, 1D, 1R, 1AB)
irf7.L (XM_018257597)	1.097	**29.2**	32/7 (16C, 7AF, 4AB, 2I…)
irf8.L (NM_001093628)	3.000	10.0	30/9 (10C, 8D, 2E, 2R…)
irf8.S (XM_018260595)	0.489	4.1	2/2 (1C, 1R)
irf9.L (NM_001091377)	1.474	11.5	17/6 (8C, 3R, 3AF…)
irf10.L (XM_018235039)	0.587	**63.0**	37/9 (17C, 4I, 3D, R3…)
socs1.L (NM_001159688)	0.353	0.0	0
socs1.S (NM_001092026)	0.355	11.3	4/4 (1D, 1E, 1L, 1AB)
	Ave: 0.862	Ave: 19.7	

Acc., accession; Ave., average; kb, kilobase; UTR, untranslated region. *Numbers higher than the average are bold.

**Figure 9:**
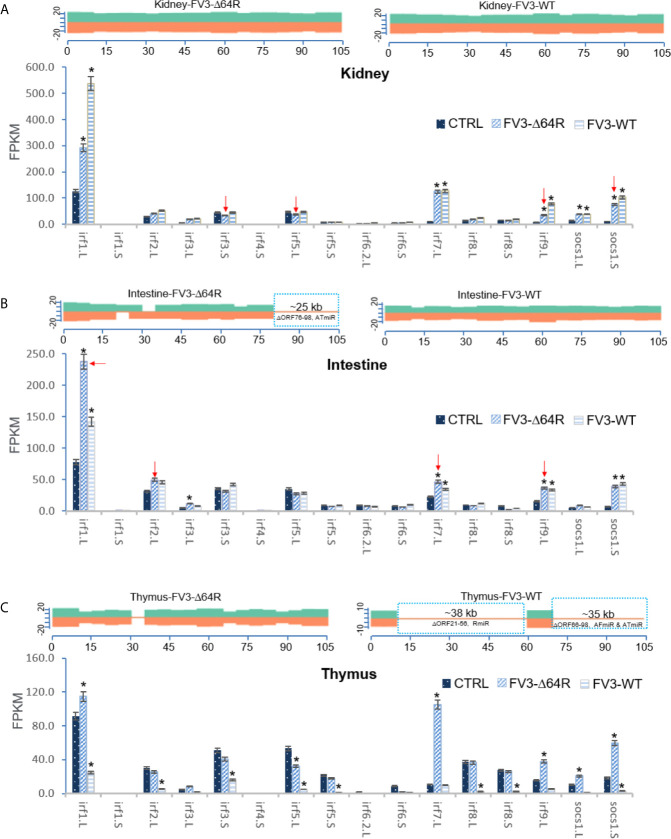
Transcriptomic comparison of the viral genome and *X. laevis* mRNA encoding interferon IFN regulatory factors (irf) in the mock, FV3-Δ64R and FV3-WT infected kidney **(A)**, intestine **(B)**, and thymus **(C)**. The distribution plots of mapped reads alone FV3 genome (GenBank Accession No. NC_005946.1) is shown as in **Figure 3** with a full-genome coverage for the infected kidney samples. Partial coverages of the viral genome were determined in the FV3-Δ64R infected intestine and for both FV3-WT and FV3-Δ64R in the infected thymus. Comparative alignments indicates that transcripts of some ORFs and miRNA-enriched intergenic regions are defective (labeled and framed using blue line) as compared between two virus strains. Analysis shows reduced expression of IRF genes corresponding to potential higher expression of respective miRNA by FV3-Δ64R in kidney and FV3-WT in intestine (indicated by red arrows). However, miRNA-mediated reduction of IRF genes is not detected in FV3-Δ64R infected thymus. This suggests a tissue- and virus strain-dependent expression of miRNA and interference on host gene targets. Abbreviations and gene accession numbers are listed in **Table 1**. *p (FDR) < 0.05 relative to the control.

As presented above, virus-focused transcriptomic analysis has revealed a genome-wide coverage for RNA-Seq reads in FV3 infected kidney samples. The study has also revealed a partial coverage of deficient FV3 strain FV3-Δ64R in infected intestine as well as both WT-FV3 and Δ64R infected thymus. Comparative alignments showed that transcripts of some ORFs and miRNA-enriched intergenic regions were lacking. Comparative gene profiling further indicates reduced expression of some *Xenopus* IRF genes, which appears to correlate with a higher expression of respective v-miRs by FV3-Δ64R in kidney and FV3-WT in intestine (**Figures 9A, B**). However, as for IFN receptor genes examined above, this putative v-miR-mediated repression system of IRF genes was not consistently detected in FV3-Δ64R-infected thymus (**Figure 9C**). This suggests a tissue- and virus strain-dependent expression of ranaviral v-miRs and a distinct interfering effect on certain host gene targets. Further studies will screen most effective v-miRs, characterize their tissue expression patterns during viral infection, and use synthetic miRNA to validate their function in modulation of host genes critically mediating amphibian IFN-dependent antiviral immunity (44, 45, 48, 49).

**Figure 10:**
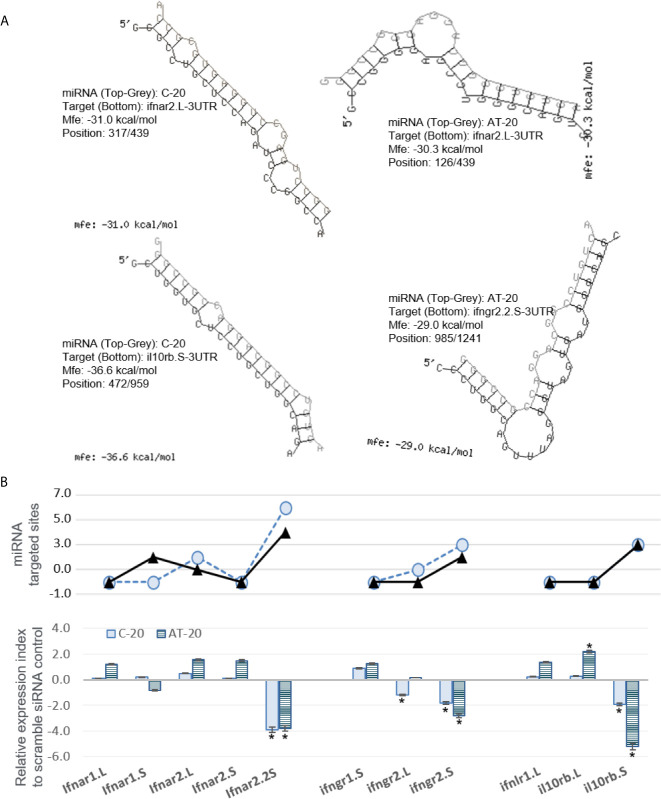
Examples of miRNA that are predictably targeted on 3’-UTR regions of *Xenopus* mRNA encoding interferon receptor subunits. **(A)** Hybridization of individual miRNA with its mRNA targets was performed using a program of RNAhybrid with its accompanying programs RNAcalibrate and RNAeffective as described. The hybridization structures and minimum free energy (Mfe) are given, the thresholds of Mfe was set as -28.0 kcal/mol to reflect typical Mfe of miRNA (like *let7*) hybridization to mRNA targets. MiRNA C-20 or AT-20, the twentieth miRNA in the C or AT groups, respectively, as illustrated in **Figure 2** and **Supplement Excel sheet** for sequence detail. **(B)** Functional validation using synthetic siRNA with identical sequences to the mature C-20 and AT-20 miRNAs. Synthesis of siRNAs and transfection of *X. laevis* A6 cells were performed as described, and gene specific RT-PCR was used to quantify the expression of target genes. *Top panel*: Line chart representing numbers of predicted sites targeted by miRNA on the 3’-UTR of each template target. *Bottom panel*: Bar chart of relative gene expression obtained with mature C-20 (gray histogram) and AT-20 (hachured histogram) miRNAs. The GenBank Accession numbers of the tested transcripts are listed in **Table 2**. *p < 0.05, n = 5 relative to the sample transfected using a scramble siRNA.

**Figure 11:**
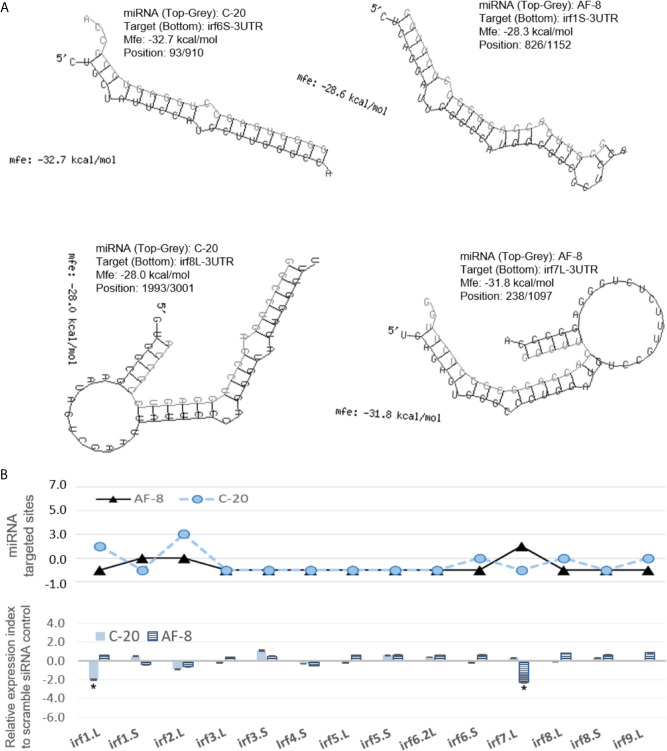
Examples of miRNA that are predictably targeted on 3’-UTR regions of X. laevis mRNA encoding several IRF genes. **(A)** Hybridization of individual miRNA with its mRNA targets was performed using a program of RNAhybrid with its accompanying programs RNAcalibrate and RNAeffective as described. The hybridization structures and minimum free energy (Mfe) are given, the thresholds of Mfe was set as -28.0 kcal/mol to reflect typical Mfe of miRNA (like let7) hybridization to mRNA targets. MiRNA C-20 and AF-8, the twentieth and eighth miRNA in the C and AF group, respectively, as illustrated in **Figure 2** and **Supplement Excel sheet** for sequence detail. **(B)** Schematic shows validation using synthetic siRNA with identical sequences to representative miRNAs. Synthesis of siRNAs and transfection of X. laevis A6 cells were performed as described, and gene specific RT-PCR was used to quantify the expression of target genes. *Top panel*: Line chart representing numbers of predicted sites targeted by miRNA AF-8 (black triangles) and C-20 (blue cicles) on the 3’-UTR of each template target. *Bottom panel*: Bar chart of relative gene expression obtained with C-20 (blue histogram) and AF-8 (hachured histogram) miRNAs. The GenBank Accession numbers of the tested transcripts are listed in **Table 2**. *p < 0.05, n = 5 relative to the sample transfected using a scramble siRNA.

Next, we sought to validate the functional effect of exemplary v-miRs. As shown in **Figures 10** and **11**, we first examined the hybridization characteristics of individual miRNA with its mRNA targets, especially of those within the 3-UTR of predicted *Xenopus ifnxr2* or *irf* genes (**Figures 10A** and **11A**). For most predicted v-miRs, their hybridization structures and minimum free energy (Mfe) to the targeted *ifnxr* and *irf* genes were found alike to at least one characterized miRNA in the miRNA database (http://www.mirbase.org/). Indeed, the threshold of Mfe for the v-miR prediction was set as -28.0 kcal/mol to reflect Mfe of typical miRNA (like *let7*) hybridization to its mRNA target. **Figure 10A** demonstrates the hybridization position, secondary structure and Mfe of v-miR C-20 or AT-20 to interact with *ifnxr2* gene targets at one site of each gene; and **Figure 11A** shows these hybridization characteristics of v-miR C-20 and AF-8 to *irf* genes. Noted that some v-miR has multiple target sites on the targeted genes, such as both C-20 and AT-20 have six targeting sites on the 3-UTR of *ifnar2.2S*, and have three or four target sites on *il10rb.S*, respectively (**Figure 10B**, line chart). Using synthetic siRNA with identical sequences to the mature C-20 and AT-20 miRNAs, we showed that the relative expression level of individual *Xenopus infxr* genes in the siRNA-transformed *X. laevis* kidney cells, were reversely correlated to the numbers of targeted sites on respective gene 3-UTR. Similar was the siRNA mimicking C-20 or AF-8 in suppression of *irf* genes in **Figure 11B**. This was with the exceptions, such as limited suppressive effect of AT-20 on *ifnar2.L*, indicating varied RNA silence effect of relevant v-miRs per each targeted gene. Therefore, in addition to the transcriptomic data to show active transcription of FV3’s intergenic v-miRs, the suppression on targeted IFN receptor and IRF genes using sequence-identical siRNAs in *Xenopus* kidney cells provides a model for functional verification of these newly identified v-miRs along a ranavirus genome (43–45).

## Conclusive Highlights

In the present study, we characterized the whole transcriptome of Frog Virus 3 (FV3), a representative Ranaviruses that causes prevalent infection in anurans and is implicated in catastrophic amphibian declines (1–7). We focused our analysis on transcription activity of FV3 non-coding intergenic regions to infer their potential regulatory role. We detected significant levels of virus-specific reads from non-coding intergenic regions distributed genome-wide, in addition to those highly in coding genes as previously reported (27). Further analyses identified various *cis*-regulatory elements (*CREs*) in these intergenic regions corresponding to transcriptomic profiles of highly expressed coding genes. These *CREs* include not only the TATA-Box-like similar to *bona fide* TATA-Box marking the core promoters of typical eukaryotic genes, but also viral mimics of *CREs* interacting with various transcription factors including CREBs, CEBPs, IRFs, NF-κB, and STATs, which are all critical for regulation of cytokine responses and cellular immunity (18, 37–42). In addition, we provide evidence suggesting that intergenic regions immediately upstream of highly expressed FV3 genes have evolved to enhance targeting and silencing IRFs, NF-κB, and STATs. Moreover, for the first time in a ranavirus, we reveal the enrichment of putative microRNA sequences in more than five intergenic regions of FV3 genome. An array of these virus-derived miRNAs is predicted to target the 3’-UTR regions of *Xenopus* genes involved in IFN-dependent immune responses, notably those encoding IFN receptor subunits and IFN-regulatory factors (39, 40, 57). Using the FV3 model, this study provides the first genome-wide analysis of non-coding regulatory mechanisms in ranaviruses *in vivo*. As such, this study contributes to a better understanding of the coevolution of epigenetic regulation viral and host gene expressions, especially centered on the host IFN system (27, 32, 33, 57).

## Data Availability Statement

The datasets presented in this study can be found in online repositories. The names of the repository/repositories and accession number(s) can be found in the article/**Supplementary Material**.

## Ethics Statement

The animal study was reviewed and approved by University of Rochester Committee on Animal Resources (UCAR) regulations (approval number 100577/2003-151).

## Author Contributions 

YT, CNK, JL, and FJ contributed to conduct experiments, student training, and proof reading. JR contributed to conceptualization, funding acquisition, advisory direction, and resource sharing. YS performed overall conceptualization, experimental coordination, data analysis, draft writing, and funding acquisition. All authors contributed to the article and approved the submitted version.

## Funding

This work was primarily supported by NSF-IOS-1831988, and in part through reagent sharing of USDA NIFA Evans-Allen-1013186, NIFA AFRI 2018-67016-28313, and NIFA AFRI 2020-67016-31347 to YS, and NSF-IOS-1456213 to JR. The Xenopus laevis research resource for immunobiology is supported by NIH R24AI059830.

## Conflict of Interest

The authors declare that the research was conducted in the absence of any commercial or financial relationships that could be construed as a potential conflict of interest.
